# Country differences in the cross-sectional associations between smoking and depressive symptoms in adolescence

**DOI:** 10.1093/eurpub/ckac155

**Published:** 2022-11-04

**Authors:** Elena Raffetti, Francesco Donato, Federico Triolo, Filip Andersson, Yvonne Forsell, Maria Rosaria Galanti

**Affiliations:** Department of Global Public Health, Karolinska Institutet, Stockholm, Sweden; Centre of Natural Hazards and Disaster Science, Uppsala University, Uppsala, Sweden; Heart and Lung Research Institute, University of Cambridge, Cambridge, UK; British Heart Foundation Cardiovascular Epidemiology Unit, Department of Public Health and Primary Care, University of Cambridge, Cambridge, UK; Department of Medical and Surgical Specialties, Radiological Sciences and Public Health, Unit of Hygiene, Epidemiology and Public Health, University of Brescia, Brescia, Italy; Department of Neurobiology, Care Sciences and Society, Aging Research Center, Karolinska Institutet, Stockholm, Sweden; Department of Global Public Health, Karolinska Institutet, Stockholm, Sweden; Centre for Epidemiology and Community Medicine, Stockholm Health Care District, Stockholm Region, Stockholm, Sweden; Department of Global Public Health, Karolinska Institutet, Stockholm, Sweden; Centre for Epidemiology and Community Medicine, Stockholm Health Care District, Stockholm Region, Stockholm, Sweden; Department of Global Public Health, Karolinska Institutet, Stockholm, Sweden; Centre for Epidemiology and Community Medicine, Stockholm Health Care District, Stockholm Region, Stockholm, Sweden

## Abstract

**Background:**

The aim of the present study was to compare the cross-sectional association between smoking and depressive symptoms among adolescents between Sweden and Italy, two countries historically characterized by different norms about tobacco use and different tobacco control efforts.

**Methods:**

A cross-sectional study including 3283 adolescents 15–16 years of age participating in the Swedish KUPOL study and 1947 same-age adolescents from the Italian BE-TEEN study. Current smoking was defined as any smoking in the past 30 days. Depressive symptoms were assessed using the Centre for Epidemiological Studies Depression Scale for Children (CES-DC) and the internalizing score of the Strengths and Difficulties Questionnaire (SDQ). Country differences were explored in stratified and interaction analyses.

**Results:**

Current smoking was associated with a 2- to 3-fold increased odds of depressive symptoms among Swedish adolescents using both CES-DC and SDQ internalizing scale. Among Italian adolescents, slightly lower increased odds of 1.5–2.5 for depressive symptoms with smoking were found using the CES-DC but not the SDQ scale. Both multiplicative and additive interactions for country were significant. The association between smoking and depressive symptoms was weaker among Italian compared with Swedish adolescents for both scores.

**Conclusions:**

Countries with different tobacco norms and control show different associations between smoking and depressive symptoms in adolescence, probably due to different psychosocial profiles of smokers. These findings need to be considered when planning tobacco prevention programmes, e.g. by focusing on early detection of mental health distress among adolescents in settings with declining smoking prevalence and restrictive tobacco control environments.

## Introduction

Epidemiological research has indicated a robust association between cigarette smoking and depressive symptoms in both adolescents and adults.^1^ This co-occurrence of smoking behaviour and depressive symptoms may be interpreted as an effect of different causal mechanisms. For example, subjects with depressive symptoms may cope with their symptoms by smoking (self-medication). Conversely, prolonged exposure to tobacco nicotine may dysregulate neuroendocrine systems and dopaminergic pathways,^2^ leading to the onset of depressive symptoms.^3^ Finally, common liability to both substance use and depressive symptoms may contribute to this relationship.^4^

Clarifying in *where*, *when* and *for whom* the smoking–depression association occurs is a key component for the understanding of possible causal mechanisms.^5^ In particular, it would be important to study how societal norms and values along with economic well-being may affect the association between smoking behaviour and depressive symptoms. For example, country-level tobacco policies are known to strongly influence smoking behaviour directly through regulations (smoke-free air policies), economic instruments (cigarette taxation/price) and interventions to reduce tobacco consumption (tobacco control funding).^6^^,^^7^ Furthermore, restrictive tobacco policies could also impact people’s behaviour indirectly, by reducing the perceived social acceptability of smoking.^8^

Among the Member States of the European Union, Italy and Sweden have been characterized by great differences in the prevalence of cigarette smoking. The Swedish society, compared with the Italian one, has been characterized by a lower acceptance of smoking, a widespread communication of its risks and stricter tobacco control measures.^9^ Starting from 1993, the Swedish governments have implemented several tobacco control measures making Sweden a restrictive smoking environment also envisioning the social unacceptability of smoking. These measures were followed by a steady decline in the prevalence of daily smoking among adults, now close to 7%, the lowest in Europe.^9^ Although the Italian government banned smoking in all indoor public places 5 months before the Swedish one (January vs. June 2005), and tobacco tax rate on cigarette retail price has been slightly higher in Italy than in Sweden (75.9% vs. 68.5%),^10^ the Italian smoking downtrend has levelled off at 25.0% prevalence of adult daily smoking in 2016–19, i.e. three times as high as in Sweden.^9^^,^^11^ Factors potentially contributing to this sustained high prevalence are the relatively recent intensification of tobacco control measures, along with the insufficient funding of tobacco prevention and cessation programmes by the Italian government. Only 10 million euros per year have been allocated to these activities compared with the 40 million euros in Sweden,^10^ a country with a 6-fold smaller population.

On the other end, Italy and Sweden are similar in relation to the prevalence of depression and the number of age-standardized years lost attributable to depression (1113–1180.7 per 100 000).^12^

**Figure 1:**
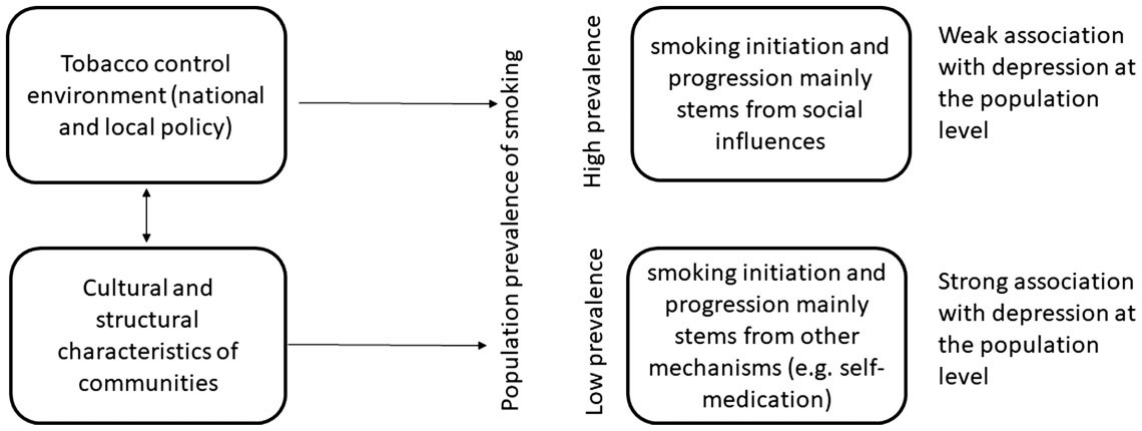
The framework describes the relationship between tobacco control environment, cultural and structural characteristics of communities, prevalence of smoking behaviour in the general population and in turn the strength of the smoking–depressive symptoms association

Tobacco control, along with cultural and structural characteristics of a community, influence the prevalence of smoking in the society. It might be hypothesized that a society with a low prevalence of smoking behaviours (more restrictive tobacco control environment) will prevent the uptake of smoking among individuals who would initiate because of social modelling, rather than among individuals with strong vulnerability to substance use and concurrent mental health problems. Along with this, societal disapproval of smoking behaviour may increase feelings of social exclusion and inadequacy among smokers. This would result in a stronger smoking–depressive symptoms association among the comparatively fewer users in restrictive tobacco control environments than in liberal tobacco control environments (figure 1 and Supplementary figure S1). Such a mechanism may be apparent in the comparison between Sweden where, along with a decrease in prevalence, there has been notable segregation of smoking among individual with psycho-social disadvantage, and Italy where this process has not yet been observed.

Adolescence may be instrumental to untangle the smoking–depressive symptoms association as it represents a critical period for both cigarette smoking initiation and the onset of depressive symptoms.^13^ In particular, during this period, multiple-level influences, including broader sociocultural factors and community norms may shape individuals’ lifestyle behaviours and affect depression susceptibility.^14^

Little is known on how country-level differences in tobacco control environment and in the cultural and structural characteristics of communities may affect on the association between smoking and depressive symptoms. This study aimed to examine these differences using observational data accrued in two countries with distinct tobacco control environments and prevalence of smoking in the general population. Because studies indicated that females might be more sensitive to depression liability^15^ and cigarette smoking dependence,^16^ the study also aimed to understand gender differences in the country-specific associations.

## Methods

This was a cross-sectional study using data from self-administered questionnaires collected within the Swedish KUPOL study and the Italian BE-TEEN study. The study was approved by the Ethics Review Board of Stockholm Region in Sweden (Dnr: 2012/1904-31/01) and Brescia Province in Italy (Dnr: 2761-04-10-2017). All parents or legal guardians completed a written informed consent in the KUPOL study and the longitudinal part of the Italian BE-TEEN study, while no consent was requested for the Italian BE-TEEN anonymous survey. Non-monetary incentives were provided for participating.

### The Swedish KUPOL study

The KUPOL study is a prospective study with the aim to explore the relationship between academic achievements/school climate and mental health problems in Swedish adolescents. The details of the KUPOL study have been published elsewhere.^17^ The following inclusion criteria were applied: schools with at least 20 students from the seventh to ninth grade located in eight regions of southern/central Sweden and students without severe learning disabilities and with an understanding of the Swedish language. Five-hundred and forty-one schools in eight regions of Sweden were invited to participate, and of these 101 (19%) were eligible and participated in the study and 3671 students answered the baseline questionnaire (original sample), yielding a 29% participation rate at the individual level. Participants with parents who had a university education and a high socio-economic status were overrepresented compared with the general Swedish student population.

Baseline questionnaire data were collected in the 2013–14 and 2014–15 school years (two separated sub-cohorts). Students and parents who accepted were followed-up every year for the subsequent 3 years.

### The Italian BE-TEEN study

The BE-TEEN study was designed to conduct a cross-country comparison (Italy and Sweden) of the relationship between lifestyles and mental health problems with inclusion criteria as in the KUPOL study. The study area was the Province of Brescia, located in Northern Italy. All 39 public schools of the Province area were contacted, of these 15 (38.5%) participated. Eligible students (*n* = 2316) in the second grade of the Italian high school (15–16 years of age) were invited to participate in an anonymous survey, of whom 2166 participated (93.6%) in the academic year 2017–18. Concurrently to the survey, a longitudinal study was initiated including students in the first grade of the Italian high school (14–15 years of age), who were followed-up every year for the subsequent 4 years. Therefore, the longitudinal and cross-sectional samples of the BE-TEEN study are composed of different individuals. The same student questionnaire used in the KUPOL study was used in the BE-TEEN study, translated in Italian using a back-translation approach. Students answered using a paper or a web-based version during school time.

### Analytical samples

To conduct the analysis on comparable age groups (15–16 years of age), we used data from participants in the BE-TEEN cohort study in the ninth grade of Swedish school (*n* = 3351) and the anonymous survey of the Italian BE-TEEN study (*n* = 1962). Participants with missing data on smoking behaviour and/or mental ill-health scores were excluded (*n* = 83). The final sample encompassed 3283 Swedish and 1947 Italian students. All students had the same age and enrolled in the same grade (compulsory schooling year for both Italian and Swedish systems).

To verify if differences in the recruitment process, i.e. active recruitment in Sweden vs. anonymous survey in Italy, affected the selection of participants and the study estimates, we compared the Swedish sample with the longitudinal sample of the BE-TEEN study at the second follow-up (*n* = 772).

### Variables

Self-reported information was used to assess cigarette smoking and dependence. Students were categorized as current smokers if they reported cigarette smoking in the past 30 days. Perceived dependence from cigarette smoking was assessed among ever smokers and categorized as present if a positive answer was given to the question: ‘Did you ever feel you are/were addicted to tobacco?’; absent otherwise.

We evaluated the role of country and gender as effect modifiers. ‘Country’ was a binary variable (Sweden/Italy).

Depressive symptoms severity was assessed using the Swedish and Italian versions of the Centre for Epidemiological Studies Depression Scale for Children (CES-DC), a 20-item scale for depressive disorders in young people aged 6–17 years.^18^ The CES-DC score was considered both as a continuous and as a binary variable, applying a cut-off score ≥ 30 as suggestive of depressive symptoms.^19^ Moreover, we evaluated depressive mood using the internalizing score of the Strengths and Difficulties Questionnaire (SDQ).^20^ The emotional and peer problems SDQ subscales were summed up using a cut-off score ≥ 9 as suggestive of internalizing problems.^21^

Parental education was included as a potential confounder and was obtained from the parent questionnaire for Swedish students and from the student questionnaire for Italian students.

### Statistical analysis

Characteristics of participants were stratified by country and expressed as means or percentages as appropriate. Logistic regression models were used to assess the association between smoking phenotypes and depressive symptoms. Results were expressed in terms of odds ratio (OR) and 95% confidence interval (95% CI). Models were also adjusted for parental education as a potential confounder. Effect modification by country was investigated through stratified analyses along with formal analyses of both multiplicative and additive interactions.^22^ Multiplicative interactions were assessed using the following formula: OR_11_/(OR_10_ × OR_01_), (OR_11_ defined as odds ratio of the joint effect of smoking behaviour and Italy as country). The Relative Excess Risk Due to Interaction (RERI) (RERI = OR_11_ − OR_10_ – OR_01_ + 1) and corresponding standard errors (delta method) were estimated to analyze additive interaction.^5^ All the analyses were also stratified by gender to evaluate its role as an additional effect modifier.

To ascertain whether differences in the recruitment process and participant selection impacted on interaction estimates, we conducted a sensitivity analysis using data from the longitudinal sample of the Italian BE-TEEN study. All analyses were carried out with Stata software version 14.0 (StataCorp, LP).

## Results

Smoking behaviour and alcohol consumption were more common among Italian compared with Swedish students (smoking: 32.3 vs. 7.3%, alcohol: 6.7% vs. 43.5%) (Supplementary table S1). Depressive symptoms were also slightly more frequent among Italians than Swedes for both CES-DC (18.4% vs. 15.2%) and SDQ (24.7% vs. 17.0%) scale. Within each country, the prevalence of current cigarette smoking did not vary according to parental education, while the prevalence of perceived smoking dependence among ever smokers was slightly higher in the low parental education group (low vs. high education: 30.1% vs. 22.6% in Italy and 36.4% vs. 22.1% in Sweden) (Supplementary table S2).

Table 1 reports the prevalence of depressive symptoms in relation to smoking stratified by country. In both countries, the prevalence of depressive symptoms was higher among current smokers compared with non-smokers, particularly among Swedish adolescents. In Sweden, 33.9% of current smokers reported depressive symptoms compared with 26.3% of current smokers in Italy, while the prevalence of depressive symptoms among non-smokers was about 14.0% for both countries and this was consistent among genders. For internalizing symptoms, the difference between smokers and non-smokers was quite pronounced in Sweden and absent in Italy (Supplementary table S3).

**Table 1:** Prevalence of depressive symptoms (CES-DC ≥ 30, binary variable) according to cigarette smoking/dependence by country and sex

	**CES-DC score above threshold**	
	Sweden *n*/total (%^a^)	Italy *n*/total (%^a^)
**Current cigarette smoking**		
Both genders		
Total	495/3266 (15.2)	323/1754 (18.4)
Yes	81/239 (33.9)	152/579 (26.3)
No	414/3027 (13.7)	171/1175 (14.6)
Females		
Total	419/1706 (24.5)	253/955 (26.5)
Yes	69/154 (44.8)	127/326 (39.0)
No	350/1552 (22.5)	126/629 (20.0)
Males		
Total	75/1559 (4.8)	70/799 (8.8)
Yes	12/85 (14.1)	25/253 (9.9)
No	63/1474 (4.3)	45/546 (8.2)
**Perceived tobacco dependence** ^b^		
Both genders		
Total	147/534 (27.5)	205/861 (23.8)
Yes	53/136 (39.0)	78/236 (33.1)
No	94/398 (23.6)	127/625 (20.3)
Females		
Total	125/313 (39.9)	166/475 (35.0)
Yes	44/83 (53.0)	66/145 (45.5)
No	81/230 (35.2)	100/330 (30.3)
Males		
Total	22/221 (10.0)	39/386 (10.1)
Yes	9/53 (17.0)	12/91 (13.2)
No	13/168 (7.7)	27/295 (9.1)

a Row percentages.

b Among ever smokers.

CES-DC: Centre for Epidemiological Studies Depression scale for Children.

Cross-sectional ORs of depressive symptoms (CES-DC scale) in relation to smoking behaviour/perceived dependence are presented in table 2. Current cigarette smoking along with perceived smoking dependence were associated with two times higher odds of depressive symptoms among both Italian and Swedish students. Adjusted models showed positive associations, however weaker, in the same direction. A separate analysis by gender revealed no association among Italian males. The interaction between smoking behaviour and country was significant. In particular, the association between current smoking and depressive symptoms was significantly weaker among Italian students compared with Swedish ones on both additive [RERI: –1.06 (–2.06 to –0.06)] and multiplicative scale [0.65 (0.44–0.94)]. This interaction effect was particularly evident among males [additive scale (RERI): –2.24 (–4.91 to 0.43) and multiplicative scale: 0.33 (0.14–0.77)] (table 3). The association of perceived smoking dependence with depressive symptoms was similar among Swedish and Italian students (no interaction).

**Table 2:** Odds ratios and 95% CIs of depressive symptoms (CES-DC) according to cigarette smoking/dependence by country and sex

	Sweden		Italy	
	Unadjusted models	Adjusted models	Unadjusted models	Adjusted models
	ORs (95% CIs)	ORs (95% CIs)	ORs (95% CIs)	ORs (95% CIs)
**Current cigarette smoking (yes vs. no)**				
Both genders	3.24 (2.43–4.31)	2.98 (2.19–4.06)	2.09 (1.63–2.67)	2.10 (1.63–2.70)
Females	2.79 (1.99–3.91)	2.60 (1.81–3.73)	2.55 (1.89–3.43)	2.56 (1.90–3.46)
Males	3.68 (1.90–7.13)	3.20 (1.51–6.78)	1.22 (0.73–2.04)	1.24 (0.73–2.09)
**Perceived tobacco dependence^a^ (yes vs. no)**				
Both genders	2.07 (1.36–3.13)	1.76 (1.13–2.75)	1.94 (1.39–2.70)	1.89 (1.35–2.66)
Females	2.08 (1.25–3.45)	1.75 (1.01–3.00)	1.92 (1.28–2.87)	1.89 (1.26–2.84)
Males	2.44 (0.98–6.08)	2.63 (0.96–7.21)	1.51 (0.73–3.11)	1.61 (0.77–3.36)

Note: Models adjusted for parental education.

a Among ever smokers.

CI: confidence intervals; CES-DC: Centre for Epidemiological Studies Depression scale for Children; OR: odds ratio.

**Table 3:** Country (Italy vs. Sweden) effect modification on the association between cigarette smoking/dependence and depressive symptoms (CES-DC)

		**Sweden** ORs (95% CIs)	**Italy** ORs (95% CIs)	ORs (95% CIs) for country within strata of cigarette smoking^a^	Effect modification on additive scale RERI (95% CI)^a^	Effect modification on multiplicative scale ORs (95% CI)^a^
	**Current cigarette smoking**					
Both genders	No	1.0	1.07 (0.89–1.30)	1.07 (0.89–1.30)	–1.06 (–2.06 to –0.06)	0.65 (0.44–0.94)
	Yes	3.24 (2.43–4.31)	2.25 (1.82–2.78)	0.69 (0.50–0.96)		
Females	No	1.0	0.86 (0.68–1.08)	0.86 (0.68–1.08)	–0.46 (–1.49 to 0.58)	0.91 (0.58–1.43)
	Yes	2.79 (1.99–3.91)	2.19 (1.70–2.82)	0.79 (0.53–1.16)		
Males	No	1.0	2.01 (1.35–2.99)	2.01 (1.35–2.99)	–2.24 (–4.91 to 0.43)	0.33 (0.14–0.77)
	Yes	3.68 (1.90–7.13)	2.46 (1.51–3.98)	0.67 (0.32–1.39)		
	**Perceived tobacco dependence^b^**					
Both genders	No	1.0	0.82 (0.61–1.12)	0.82 (0.61–1.12)	–0.29 (–1.19 to 0.61)	0.94 (0.55–1.60)
	Yes	2.07 (1.36–3.13)	1.60 (1.12–2.28)	0.77 (0.50–1.20)		
Females	No	1.0	0.80 (0.56–1.14)	0.80 (0.56–1.14)	–0.34 (–1.44 to 0.76)	0.93 (0.48–1.77)
	Yes	2.08 (1.25–3.45)	1.54 (1.01–2.35)	0.74 (0.43–1.27)		
Males	No	1.0	1.20 (0.60–2.40)	1.20 (0.60–2.40)	–0.83 (–3.19 to 1.53)	0.62 (0.19–1.98)
	Yes	2.44 (0.98–6.08)	1.81 (0.79–4.15)	0.74 (0.29–1.90)		

a Sweden considered as reference.

b Among ever smokers.

CES-DC: Centre for Epidemiological Studies Depression scale for Children; OR: odds ratio; CI: confidence intervals; RERI: Relative Excess Risk Due to Interaction.

Current cigarette smoking was related to internalizing symptoms of the SDQ scale only among Swedish students, while perceived smoking dependence was associated with higher internalizing symptoms in both Swedish and Italian students, particularly among males (Supplementary table S4). The association of cigarette smoking with SDQ internalizing score was weaker among Italian than Swedish students in the whole cohort [additive scale (RERI): –0.90 (–1.60 to –0.19) and multiplicative scale 0.53 (0.36–0.77)], mainly driven by males (only on the multiplicative scale: 0.41 (0.19–0.86)] (Supplementary table S5). No interaction was found between perceived smoking dependence and country for internalizing symptoms.

The results of the sensitivity analysis comparing the Swedish KUPOL study with Italian BE-TEEN longitudinal study are shown in Supplementary table S6. The BE-TEEN study included 772 participants, of whom 41.8% males, 20.5% current smokers and 11.4% reporting depressive symptoms according to the CES-DC scale. While weaker and less precise estimates were observed, the association between smoking behaviour and depressive symptoms (CES-DC scale) remained consistent (OR= 1.43, 0.82–2.39). An interaction effect with country was identified on both additive and multiplicative scale (Supplementary table S6).

## Discussion

Findings from this large Swedish–Italian comparative study, while confirming a consistent link between smoking and depressive symptoms in both countries, suggest a role of country-level factors on the magnitude of this association. In particular, these findings indicate that smokers compared with non-smokers have a greater likelihood of perceiving depressive symptoms in Sweden compared with Italy, mainly driven by male adolescents.

These results support the hypothesis of a stronger association between smoking behaviour and depressive symptoms in countries characterized by a restrictive tobacco control environment and in a more mature stage of the tobacco epidemic. It is not surprising that the adoption of several tobacco control measures in Sweden during the last three decades may have contributed to reducing the prevalence of smoking but also to a segregation of the behaviour within more vulnerable adolescent groups.^9^ In support of the initial hypothesis, these findings pointed towards a stronger association in Sweden compared with Italy among males, whose onset of smoking is mainly determined by social influences. Indeed, previous evidence suggested gender differences as determinants of smoking initiation and progression: while females seem to smoke mainly to regulate mood, males tend to approach smoking as social behavior.^23^

The role of the tobacco control environment along with cultural and structural characteristics of communities may be implicated in the association between smoking behaviour and depressive symptoms through different mechanisms. As stated above, the proportion of social smokers may decrease in a restrictive tobacco control environment, while the proportion of individuals smoking because of psychological reasons may not. Another explanation relies on the ‘on-time off-time’ hypothesis.^24^ This posits that in the presence of a risk factor in a period when its occurrence is least expected, the magnitude of the association with mental disorders might be greater. Italy and Sweden are at different stages of the tobacco epidemic.^25^^,^^26^ For instance, smoking is a common and foreseeable lifestyle—*on time—*among Italian adolescents, but it is *off-time* among Swedish adolescents. Therefore, being a cigarette smoker in Sweden may be a marker of depression, elicited through different pathways. For instance, societal disapproval of smoking behaviour may generate feelings of social exclusion and social inadequacy when adolescents compare themselves with friends and with society’s standards. At the same time, one ought to recognize that country-level factors not directly connected to tobacco control may also influence the association between smoking and depressive symptoms in adolescence, such as financial crises, individualistic/collectivist cultures along with climatic features. Moreover, the rise of mental health problems in both Italy and Sweden over the last decades may have resulted in a stronger association between smoking and depressive symptoms,^27^ in particular in an environment characterized by a simultaneous decrease of smoking prevalence.

The strength of this study rests on the use of the same survey methodology in two European countries and on the large sample size. While previous studies have investigated the smoking–depressive symptoms interplay among minorities in the same country,^28^^,^^29^ ours is the first that compares this relationship in two countries with different tobacco control and norms. The measure of interaction should be interpreted as a measure of country-level variability concerning the relationship between smoking and depressive symptoms, rather than having a causal connotation.^5^ However, there are some limitations worth mentioning, such as potential biases due to the cross-sectional design, along with country-differential selection bias or misclassification of the reported behaviours and symptoms. Given the cross-sectional design, we were unable to examine the temporal relationship between tobacco initiation and the onset of depressive symptoms over time, limiting causal inference. Possible selection bias due to the higher response rate in the Italian sample compared with the Swedish one (anonymous vs. parental consent survey) cannot be excluded. However, a sensitivity analysis, including a sample of Italian students undergoing a selection process similar to the Swedish one was largely confirmative of country-level heterogeneity. Given a different expression of depressive symptoms between males and females, the use of CES-DC scale, like other depression scales, may be biased towards identifying symptoms that are more frequent in females. A recent study pointed out that males are more likely to report alternative depressive symptoms as risk-taking behaviours, anger and irritability.^30^ In particular, since masculinity traits may impact the reports of typical symptoms of depression,^31^ a higher degree of masculinity norms among Italian compared with Swedish adolescents may lead to a possible higher non-differential misclassification of the outcome among Italian males. This, in turn, may result in a null smoking–depressive symptoms association among Italian males explaining the significant interaction by country. Finally, a difference in unmeasured social characteristics (i.e. negative life events and poor parenting) cannot be excluded.

Bringing attention on country-level factors as determinants of the smoking–depressive symptoms association has the potential to improve the understanding of this co-occurrence, and more importantly, to direct intervention efforts towards different targets. For instance, it could be suggested that preventive programmes in Italy should be directed to modify social norms and to increase compliance with the existing regulations. In Sweden, the focus would rather be on indicated prevention, i.e. be on tackling early signs of mental distress among young people, as predisposing factors to substance use. Future longitudinal studies, which describe the simultaneous evolution of the tobacco epidemic and the magnitude of the smoking–depressive symptoms association, should confirm findings from this study.

This study provides a deeper understanding of the smoking–depressive symptoms association explaining *for whom* this occurs, suggesting a stronger association in a restrictive (Sweden) compared with a permissive smoking environment (Italy). Future public health interventions on lifestyles and well-being among adolescents should consider both individual and context-level factors.

## Supplementary data

Supplementary data are available at *EURPUB* online.

## Supplementary Material

ckac155_Supplementary_Data

## Data Availability

The data underlying this article will be accessible on reasonable request to the KUPOL study’s PI (rosaria.galanti@ki.se) and the BE-TEEN study’s PI (elena.raffetti@ki.se). Tobacco control environment may modify smoking–depressive symptoms association. The strongest association was found in the more restrictive tobacco control environment. Psychosocial profile of smokers needs to be considered when planning tobacco prevention programmes.

## References

[1] Fluharty M , Taylor AE, Grabski M, Munafò MR. The association of cigarette smoking with depression and anxiety: a systematic review. Nicotine Tob Res 2017;19:3–13. 27199385 10.1093/ntr/ntw140 PMC5157710

[2] Nisell M , Nomikos GG, Svensson TH. Nicotine dependence, midbrain dopamine systems and psychiatric disorders. Pharmacol Toxicol 1995;76:157–62. 7617539 10.1111/j.1600-0773.1995.tb00123.x

[3] Munafo MR , Araya R. Cigarette smoking and depression: a question of causation. Br J Psychiatry 2010;196:425–6. 20513848 10.1192/bjp.bp.109.074880

[4] Edwards AC , Kendler KS. A twin study of depression and nicotine dependence: shared liability or causal relationship? J Affect Disord 2012;142:90–7. 22901332 10.1016/j.jad.2012.03.048 PMC3483438

[5] Vanderweele TJ. Explanation in Causal Inference: Methods for Mediation and Interaction. New York, NY: Oxford University Press, 2015.

[6] Frazer K , Callinan JE, McHugh J, et al Legislative smoking bans for reducing harms from secondhand smoke exposure, smoking prevalence and tobacco consumption. Cochrane Database Syst Rev 2016;2:CD005992. 26842828 10.1002/14651858.CD005992.pub3 PMC6486282

[7] Fichtenberg CM , Glantz SA. Effect of smoke-free workplaces on smoking behaviour: systematic review. BMJ 2002;325:188. 12142305 10.1136/bmj.325.7357.188 PMC117445

[8] Van Zundert RM , Engels RC, Van den Eijnden RJ. Adolescent smoking continuation: reduction and progression in smoking after experimentation and recent onset. J Behav Med 2006;29:435–47. 16855869 10.1007/s10865-006-9065-4

[9] Centrum för epidemiologi och samhällsmedicin. Folkhälsorapport 2019, Stockholms län, 2019.

[10] WHO. Available at: https://www.who.int/tobacco/global_report/2017/en/ (12 October 2020, date last accessed).

[11] Epicentro. Available at: https://www.epicentro.iss.it/passi/dati/fumo (5 October 2020, date last accessed).

[12] Ferrari AJ , Charlson FJ, Norman RE, et al Burden of depressive disorders by country, sex, age, and year: findings from the global burden of disease study 2010. PLoS Med 2013;10:e1001547. 24223526 10.1371/journal.pmed.1001547 PMC3818162

[13] Thapar A , Collishaw S, Pine DS, Thapar AK. Depression in adolescence. Lancet 2012;379:1056–67. 22305766 10.1016/S0140-6736(11)60871-4 PMC3488279

[14] Cairns KE , Yap MB, Pilkington PD, Jorm AF. Risk and protective factors for depression that adolescents can modify: a systematic review and meta-analysis of longitudinal studies. J Affect Disord 2014;169:61–75. 25154536 10.1016/j.jad.2014.08.006

[15] Kessler RC , McGonagle KA, Swartz M, et al Sex and depression in the National Comorbidity Survey. I: lifetime prevalence, chronicity and recurrence. J Affect Disord 1993;29:85–96. 8300981 10.1016/0165-0327(93)90026-g

[16] Sylvestre MP , Chagnon M, Wellman RJ, et al Sex differences in attaining cigarette smoking and nicotine dependence milestones in novice smokers. Am J Epidemiol 2018;187:1670–7. 29522067 10.1093/aje/kwy045

[17] Galanti MR , Hultin H, Dalman C, et al School environment and mental health in early adolescence – a longitudinal study in Sweden (KUPOL). BMC Psychiatry 2016;16:243. 27421757 10.1186/s12888-016-0919-1 PMC4947256

[18] Fendrich M , Weissman MM, Warner V. Screening for depressive disorder in children and adolescents: validating the Center for Epidemiologic Studies Depression Scale for Children. Am J Epidemiol 1990;131:538–51. 2301363 10.1093/oxfordjournals.aje.a115529

[19] Olsson G , von Knorring AL. Depression among Swedish adolescents measured by the self-rating scale Center for Epidemiology Studies-Depression Child (CES-DC). Eur Child Adolesc Psychiatry 1997;6:81–7. 9257089 10.1007/BF00566670

[20] Goodman R. Psychometric properties of the strengths and difficulties questionnaire. J Am Acad Child Adolesc Psychiatry 2001;40:1337–45. 11699809 10.1097/00004583-200111000-00015

[21] Goodman A , Lamping DL, Ploubidis GB. A reappraisal of the factor structure of the Strengths and Difficulties Questionnaire (SDQ): data from British parents, teachers and children. (Supplementary material) from when to use broader internalising and externalising subscales instead of the hypothesised five subscales on the Strengths and Difficulties Questionnaire (SDQ): data from British parents, teachers and children. J Abnorm Child Psychol 2010;38:1179–91. 20623175 10.1007/s10802-010-9434-x

[22] Knol MJ , VanderWeele TJ. Recommendations for presenting analyses of effect modification and interaction. Int J Epidemiol 2012;41:514–20. 22253321 10.1093/ije/dyr218 PMC3324457

[23] Waldron I. Patterns and causes of gender differences in smoking. Soc Sci Med 1991;32:989–1005. 2047903 10.1016/0277-9536(91)90157-8

[24] Neugarten BL. Time, age, and the life cycle. Am J Psychiatry 1979;136:887–94. 453348 10.1176/ajp.136.7.887

[25] Lopez AD , Collishaw NE, Piha T. A descriptive model of the cigarette epidemic in developed countries. Tobacco Control 1994;3:242–7.

[26] Bilano V , Gilmour S, Moffiet T, et al Global trends and projections for tobacco use, 1990-2025: an analysis of smoking indicators from the WHO Comprehensive Information Systems for Tobacco Control. Lancet 2015;385:966–76. 25784347 10.1016/S0140-6736(15)60264-1

[27] The Swedish National Board of Health and Welfare. Folkhalsorapporten. Sweden, 2014.

[28] Okeke NL , Spitz MR, Forman MR, Wilkinson AV. The associations of body image, anxiety, and smoking among Mexican-origin youth. J Adolesc Health 2013;53:209–14. 23669646 10.1016/j.jadohealth.2013.03.011 PMC4441269

[29] Repetto PB , Caldwell CH, Zimmerman MA. A longitudinal study of the relationship between depressive symptoms and cigarette use among African American adolescents. Health Psychol 2005;24:209–19. 15755235 10.1037/0278-6133.24.2.209

[30] Cavanagh A , Wilson CJ, Caputi P, Kavanagh DJ. Symptom endorsement in men versus women with a diagnosis of depression: a differential item functioning approach. Int J Soc Psychiatry 2016;62:549–59. 27335340 10.1177/0020764016653980

[31] Price EC , Gregg JJ, Smith MD, Fiske A. Masculine traits and depressive symptoms in older and younger men and women. Am J Mens Health 2018;12:19–29. 26634856 10.1177/1557988315619676 PMC5734536

