# Supplementary material for: Country differences in the cross-sectional associations between smoking and depressive symptoms in adolescence
Source: Eur J Public Health. 2022 Nov 4;32(6):913–8. doi: 10.1093/eurpub/ckac155 (PMC9713381; doi:10.1093/eurpub/ckac155)
Supplement: ckac155_Supplementary_Data [file ckac155_supplementary_data.docx]

**Supplementary Table 1.** Participants’ characteristics by country (The Swedish KUPOL study 2014-2015,2015-2016, The Italian BE-TEEN study 2017-2018).

|  | **Sweden**  **n (%)** | **Italy**  **n (%)** |
| --- | --- | --- |
| Total | 3283 | 1947 |
| Females | 1710 (52.1) | 1049 (53.9) |
| High parental education^a^ | 2082 (71.3) | 701 (37.3) |
| Current cigarette smoking | 239 (7.3) | 629 (32.3) |
| Perceived cigarette smoking dependence^b^ | 136 (25.4) | 252 (27.0) |
| Alcohol consumption ≥ once a month | 218 (6.7) | 837 (43.5) |
| Depressive symptoms, CES-DC, mean (SD) | 17.1 (11.2) | 18.1 (12.4) |
| Depressive symptoms, CES-DC ≥ 30 | 495 (15.2) | 323 (18.4) |
| Internalizing symptoms, SDQ mean (SD) | 5.1 (3.4) | 6.0 (3.9) |
| Internalizing symptoms, SDQ ≥ 9 | 553 (17.0) | 479 (24.7) |

^a^ one or more parents having university education. ^b^ among ever smokers

Abbreviations: Centre for Epidemiological Studies Depression scale for Children, CES-DC; Standard Deviation, SD, Strengths and Difficulties Questionnaire, SDQ.

**Supplementary Table 2**. Cigarette smoking / dependence by parental education and country

|  | **Low parental education** | | **High parental education**  ^a^ | |
| --- | --- | --- | --- | --- |
|  | **Sweden n (%)** | **Italy n (%)** | **Sweden n (%)** | **Italy n (%)** |
| **Total** | 838 | 1178 | 2082 | 701 |
| **Current cigarette smoking** | 65 (7.8) | 395 (33.5) | 145 (7.0) | 220 (31.4) |
| **Perceived tobacco dependence**  ^b^ | 51 (36.4) | 171 (30.1) | 71 (22.1) | 77 (22.6) |

^a^ one or more parents having university education. ^b^ among ever smokers

**Supplementary Table 3.** Prevalence of SDQ-assessed internalizing symptoms (cut-off ≥ 9, binary variable) by cigarette smoking / dependence, sex and country

|  | **internalizing SDQ** | |
| --- | --- | --- |
|  | **Sweden**  **n/total (%)** | **Italy**  **n/total (%)** |
| **Current cigarette smoking** | | |
| Both genders Total | 553/3260 (17.0) | 479/1942 (24.7) |
| Yes | 65/237 (27.4) | 158/628 (25.2) |
| No | 488/3023 (16.1) | 321/1314 (24.4) |
|  |  |  |
| Females Total | 417/1702 (24.5) | 355/1047 (33.9) |
| Yes | 52/154 (33.8) | 123/347 (35.5) |
| No | 365/1548 (23.6) | 232/700 (33.1) |
|  |  |  |
| Males Total | 135/1557 (8.7) | 124/895 (13.9) |
| Yes | 13/83 (15.7) | 35/281 (12.5) |
| No | 122/1474 (8.3) | 89/614 (14.5) |
|  |  |  |
| **Perceived tobacco dependence ^b^** | | |
| Both genders Total | 118/528 (22.4) | 230/932 (24.7) |
| Yes | 39/136 (28.7) | 78/252 (31.0) |
| No | 79/392 (20.1) | 152/680 (22.3) |
|  |  |  |
| Females Total | 95/312 (30.5) | 177/504 (35.1) |
| Yes | 31/83 (37.4) | 61/151 (40.4) |
| No | 64/229 (28.0) | 116/353 (32.9) |
|  |  |  |
| Males Total | 23/216 (10.7) | 53/428 (12.4) |
| Yes | 8/53 (15.1) | 17/101 (16.8) |
| No | 15/163 (9.2) | 36/327 (11.0) |

^b^ among ever smokers

Abbreviation: Strengths and Difficulties Questionnaire, SDQ.

**Supplementary Table 4.** Odds ratios and 95% CI of incidence of SDQ-assessed internalizing symptoms according to cigarette smoking / dependence by country and sex.

|  | **Sweden** |  |  | **Italy** |  |
| --- | --- | --- | --- | --- | --- |
|  | **Unadjusted models**  ORs (95% CIs) | **Adjusted models**  ORs (95% CIs) |  | **Unadjusted models**  ORs (95% CIs) | **Adjusted models**  ORs (95% CIs) |
| **Current cigarette smoking (yes vs no)** |  |  |  |  |  |
| Both genders | 1.96 (1.45-2.65) | 1.85 (1.34-2.57) |  | 1.04 (0.83-1.30) | 1.03 (0.82-1.28) |
| Females | 1.65 (1.16-2.35) | 1.55 (1.06-2.27) |  | 1.11 (0.85-1.45) | 1.10 (0.84-1.44) |
| Males | 2.06 (1.11-3.83) | 2.02 (1.03-3.96) |  | 0.84 (0.55-1.28) | 0.83 (0.54-1.28) |
|  |  |  |  |  |  |
| **Perceived tobacco dependence ^a^ (yes vs no)** |  |  |  |  |  |
| Both genders | 1.59 (1.02-2.49) | 1.43 ( 0.88-2.32) |  | 1.56 (1.13-2.15) | 1.49 (1.07-2.07) |
| Females | 1.54 (0.90-2.61) | 1.41 (0.80-2.50) |  | 1.38 (0.93-2.05) | 1.30 (0.87-1.93) |
| Males | 1.75 (0.70-4.40) | 1.77 (0.65-4.86) |  | 1.64 (0.87-3.06) | 1.74 (0.92-3.29) |

**Models** adjusted for parental education. ^a^ among ever smokers

Abbreviation: Strengths and Difficulties Questionnaire, SDQ; Odds Ratio, OR, Confidence Intervals, CI.

**Supplementary Table 5.** Country (Italy vs. Sweden) effect modification on the association between cigarette smoking / dependence and SDQ-assessed internalizing symptoms.

|  |  | **Sweden**  ORs (95% CIs) | **Italy**  ORs (95% CIs) | ORs (95% CIs) for country within strata of cigarette smoking ^a^ | Effect modification on additive scale RERI (95% CI) ^a^ | Effect modification on multiplicative scale ORs (95% CI) ^a^ |
| --- | --- | --- | --- | --- | --- | --- |
| **Current cigarette smoking** |  |  |  |  |  |  |
| Both genders | No | 1.0 | 1.68 (1.43-1.97) | 1.68 (1.43-1.97) | -0.90 (-1.60,-0.19) | 0.53 (0.36-0.77) |
|  | Yes | 1.96 (1.45-2.65) | 1.75 (1.42-2.14) | 0.89 ( 0.63-1.25) |  |  |
|  |  |  |  |  |  |  |
| Females | No | 1.0 | 1.61 (1.32-1.96) | 1.61 (1.32-1.96) | -0.48 (-1.22,0.26) | 0.67 (0.43-1.05) |
|  | Yes | 1.65 (1.16-2.35) | 1.78 (1.39-2.28) | 1.08 (0.72-1.61) |  |  |
|  |  |  |  |  |  |  |
| Males | No | 1.0 | 1.88 (1.40-2.51) | 1.88 (1.40-2.51) | -1.36 (-2.83,0.11) | 0.41 (0.19-0.86) |
|  | Yes | 2.06 (1.11-3.83) | 1.58 (1.06-2.35) | 0.77 (0.38-1.53) |  |  |
|  |  |  |  |  |  |  |
| **Perceived tobacco dependence ^b^** |  |  |  |  |  |  |
| Both genders | No | 1.0 | 1.14 (0.84-1.55) | 1.14 (0.84-1.55) | 0.04 (-0.78,.86) | 0.98 (0.56-1.69) |
|  | Yes | 1.59 (1.02-2.49) | 1.78 (1.23-2.55) | 1.11 (0.71-1.76) |  |  |
|  |  |  |  |  |  |  |
| Females | No | 1.0 | 1.26 (0.88-1.82) | 1.26 (0.88-1.82) | \| -0.05 (-1.03,0.93) \| \| --- \| | 0.90 (0.47-1.74) |
|  | Yes | 1.54 (0.90-2.61) | 1.75 (1.13-2.70) | 1.14 (0.66-1.97) |  |  |
|  |  |  |  |  |  |  |
| Males | No | 1.0 | 1.22 (0.65-2.30) | 1.22 (0.65-2.30) | 0.02 (-1.78,-1.83) | 0.93 (0.31-2.84) |
|  | Yes | 1.75 (0.70-4.40) | 2.00 (0.95-4.20) | 1.14 (0.46-2.84) |  |  |

^a^ Sweden considered as reference ^b^ among ever smokers

Abbreviation: Strengths and Difficulties Questionnaire, SDQ; Odds Ratio, OR, Confidence Intervals, CI; Relative Excess Risk Due to Interaction, RERI.

|  |  | **Sweden**  ORs (95% CIs) | **Italy**  ORs (95% CIs) | ORs (95% CIs) for country within strata of cigarette smoking ^a^ | Effect modification on additive scale RERI (95% CI) ^a^ | Effect modification on multiplicative scale ORs (95% CI) ^a^ |
| --- | --- | --- | --- | --- | --- | --- |
| **Current cigarette smoking** |  |  |  |  |  |  |
| Both genders | No | 1.0 | 0.75 ( 0.57-0.99) | 0.75 ( 0.57-0.99) | -1.91 (-2.96,-0.86) | 0.44 (0.25-0.80) |
|  | Yes | 3.24 ( 2.43-4.31) | 1.08 (0.68-1.69) | 0.33 (0.20-0.56) |  |  |
|  |  |  |  |  |  |  |
| Females | No | 1.0 | 0.63 (0.46-0.86) | 0.63 (0.46-0.86) | -1.66 (-2.69,-0.63) | 0.43 (0.22-0.85) |
|  | Yes | 2.79 (1.99- 3.91) | 0.75 (0.45-1.27) | 0.27 (0.15-0.49) |  |  |
|  |  |  |  |  |  |  |
| Males | No | 1.0 | 0.97 (0.50-1.87) | 0.97 (0.50-1.87) | -1.54 (-4.63,1.55) | 0.59 (0.16-2.13) |
|  | Yes | 3.68 (1.90-7.13) | 2.11 (0.82-5.47) | 0.57 (0.19-1.73) |  |  |

**Supplementary Table 6.** Country (Italy vs. Sweden) effect modification on the association between cigarette smoking and depressive symptoms (CES-DC) including Italian BE-TEEN longitudinal sample.

^a^ Sweden considered as reference ^b^ among ever smokers

Abbreviation: Centre for Epidemiological Studies Depression scale for Children, CES-DC; Odds Ratio, OR, Confidence Intervals, CI; Relative Excess Risk Due to Interaction, RERI.


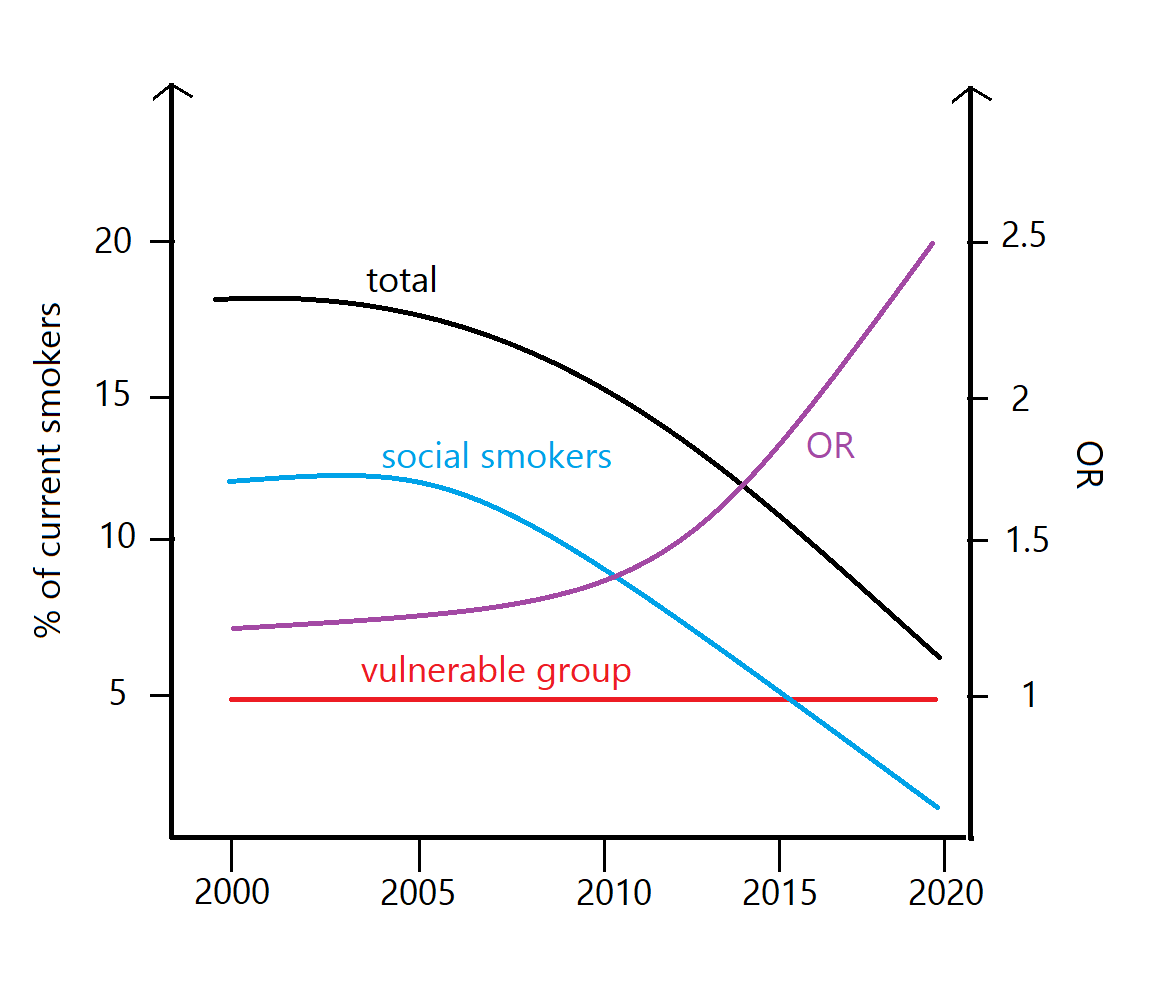


**Supplementary figure 1**. The trajectory of current smoker and of its two main components: social smokers and vulnerable group after the implementation of tobacco control measures. The OR (odds ratio) describes the magnitude of smoking-depressive symptoms association. Estimated prevalence of current smokers were derived from the Swedish general population. (Centrum för epidemiologi och samhällsmedicin. Folkhälsorapport 2019, Stockholms län. 2019).
